# Supplementary material for: AI based image analysis of red blood cells in oscillating microchannels
Source: RSC Adv. 2023 Sep 28;13(41):28576–82. doi: 10.1039/d3ra04644c (PMC10537593; doi:10.1039/d3ra04644c)
Supplement: RA-013-D3RA04644C-s003 [file RA-013-D3RA04644C-s003.pdf]

Electronic Supplementary Material (ESI) for RSC Advances.
This journal is © The Royal Society of Chemistry 2023

AI based image analysis of red blood cells in oscillating microchannels

Andreas Link,[a] Irene Luna Pardo,[a] Bernd Porr [a] and Thomas Franke*[a]

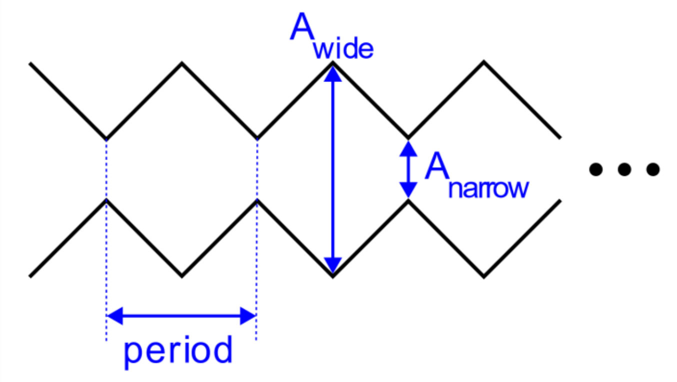

Supplemental Figure 1 The periodicity of the zigzag-channel is $L = 20$ µm *with amplitudes* $A_{wide} = 20$ µm *and* $A_{narrow} = 10$ µm. *In total are 16 periods but only the first wide and narrow section of the oscillation of the first period is used*

The Python code used in this study can be found at https://doi.org/10.5281/zenodo.7789864.
The python code which performs training, validation and testing on the RBC images. The main program is called "train.py" which can be started with "train.py -n" to use images in a narrow section or “train.py -w" on a wide section of the zigzag-shaped channel. The script will save the trained models, plots the training accuracy & loss and examples of cell classifications.
